# An ERP Study of Good Production vis-à-vis Poor Perception of Tones in Cantonese: Implications for Top-Down Speech Processing

**DOI:** 10.1371/journal.pone.0054396

**Published:** 2013-01-16

**Authors:** Sam-Po Law, Roxana Fung, Carmen Kung

**Affiliations:** 1 The University of Hong Kong, Hong Kong, SAR; 2 The Hong Kong Polytechnic University, Hong Kong, SAR; 3 Radboud University Nijmegen, Donders Institute for Brain, Cognition, and Behaviour, Nijmegen, The Netherlands; Baycrest Hospital, Canada

## Abstract

This study investigated a theoretically challenging dissociation between good production and poor perception of tones among neurologically unimpaired native speakers of Cantonese. The dissociation is referred to as the near-merger phenomenon in sociolinguistic studies of sound change. In a passive oddball paradigm, lexical and nonlexical syllables of the T1/T6 and T4/T6 contrasts were presented to elicit the mismatch negativity (MMN) and P3a from two groups of participants, those who could produce and distinguish all tones in the language (Control) and those who could produce all tones but specifically failed to distinguish between T4 and T6 in perception (Dissociation). The presence of MMN to T1/T6 and null response to T4/T6 of lexical syllables in the dissociation group confirmed the near-merger phenomenon. The observation that the control participants exhibited a statistically reliable MMN to lexical syllables of T1/T6, weaker responses to nonlexical syllables of T1/T6 and lexical syllables of T4/T6, and finally null response to nonlexical syllables of T4/T6, suggests the involvement of top-down processing in speech perception. Furthermore, the stronger P3a response of the control group, compared with the dissociation group in the same experimental conditions, may be taken to indicate higher cognitive capability in attention switching, auditory attention or memory in the control participants. This cognitive difference, together with our speculation that constant top-down predictions without complete bottom-up analysis of acoustic signals in speech recognition may reduce one’s sensitivity to small acoustic contrasts, account for the occurrence of dissociation in some individuals but not others.

## Introduction

The view that speech perception and production are closely connected is rather uncontroversial. In most models of spoken word production of a generic two-stage framework (see [1], [2] for review), the phonological representations are assumed to develop through prior exposure to spoken word forms in one’s language input, and to produce a target word, it is supposed that the phonemes activated by a selected lexical node are translated into some articulatory codes for subsequent motor programming and execution. For speech perception, the classic motor theory of speech perception maintains that speech perception and production are biologically linked and that speech perception must involve access to the motor system [3], [4], [5], although a strong version of the theory has recently been argued to be untenable [6]. Regardless, the strongest argument for the integration between speech perception and production comes from speech/language development, since “learning to speak is essentially a motor learning task” (p. 399, [7]).

Drawing on evidence from lesion studies including reported cases of acquired auditory comprehension deficits, word deafness and split brain patients, and studies employing various neuroimaging techniques and direct cortical stimulation, Hickok and colleagues [7], [8], [9], [10] put forth a dual-stream model of cortical networks underlying auditory-motor speech interface (see also [11], [12] for other hierarchical models of speech perception). The two streams, a dorsal and a ventral one, overlap in the left posterior superior temporal sulcus, which has been shown to activate for processes of speech perception and production [13]. The dorsal stream involves mapping sensory/phonological representations from the temporal-parietal-occipital junction onto articulatory-motor codes in the left frontal cortex, whereas the ventral stream maps sensory/phonological onto semantic representations along the left (or bilateral) middle temporal gyrus and inferior temporal sulcus. A related model taking an analysis-by-synthesis approach to speech processing more explicitly assumes that sensory representations underlying perception and articulatory motor representations supporting production are linked by distinctive phonetic features [14], [15]. The architecture of the dual stream model can account for double dissociation between speech perception and auditory comprehension among brain-damaged individuals (e.g. [16]), as well as between spoken language comprehension and production, such as those who cannot comprehend language (i.e. word deafness) but can express his/her ideas in spoken form [17], [18], [19], [20], and those with the opposite pattern (e.g. [21], [22], [23]).

More specific to speech perception and production of neurologically unimpaired individuals, the models we have discussed thus far predict that mismatch between production and perception may occur, but only the pattern of poor production and good perception is possible, as production involves motor programming of articulatory representations subsequent to access to sensory/phonological representations. This also means that good production must presume the existence of relevant sensory/phonological representations associated with good speech perception. Hence, good production vis-à-vis poor perception involving the same sensory/phonological form is apparently problematic to these models. One possible account for this dissociation can be found [7]. It is proposed that the property of auditory-motor interaction changes over the course of acquiring a new segment or sound sequence (e.g. word). The sensory representation of the new form plays an important role of guiding articulatory motor gesture initially. As the segment or segmental sequence becomes familiar, it will be less dependent on the sensory representation for guidance. Such a proposal would allow for dissociation between perception and production for familiar or frequently occurring items, if it is further supposed that the stability of the sensory representation may also change with increased independence between sensory and motor representations. For unfamiliar or low frequency lexical items (or pseudo words obeying the phonotactic constraints of the language), perception and production of these items are not expected to dissociate. Therefore, to better understand the nature of dissociation between good production and poor perception, one possible way is to realize the contrast of interest in a new or unfamiliar context, such as a low frequency unfamiliar word or a pseudo word.

**Figure 1:**
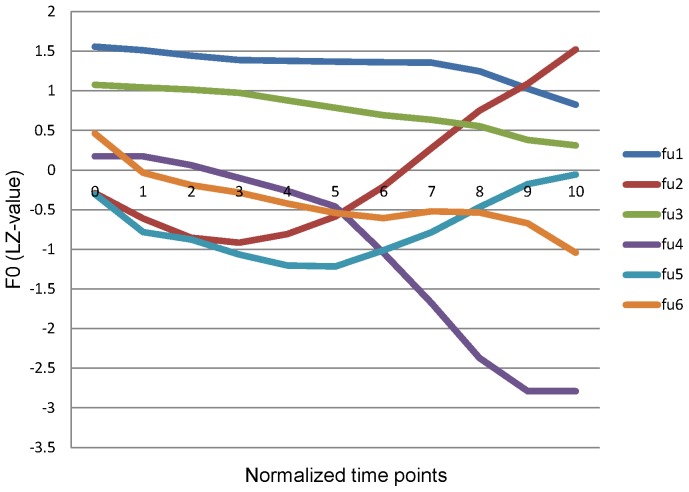
Pitch contours of time-normalized syllable *fu* in Cantonese.

In this study, we examined a dissociative pattern of good production but poor perception of speech that had been documented in sociolinguistic studies of sound change [24], [25], [26]. Labov and colleagues named this phenomenon “near-merger”. It has baffled linguists since its first description because it challenges the dominant models of phonological processing. While the early reports of near-merger are concerned with segmentals [24], [25], [26], [27], the phenomenon may also involve suprasegmentals, such as tone [28], [29]. Note that the term near-merger has also been used to describe a different phenomenon, a morphological process in which a syllable with non-high tone changes to high rising tone after fusing with a high tone diminutive morpheme [30]. The resultant form is called ‘changed tone’. Cantonese speakers have been found to have different phonetic realizations of the lexical high rising tone and the changed tone, but fail to perceive their difference [30]. Recently, an investigation of whether tone-merger exists in Cantonese, and if so how extensive it is, has been carried out in Hong Kong [31], [32]. Cantonese is a tonal language of the Chinese language family. It stands out from other tone languages in the world by having a rich system of tonal contrasts. There are six contrastive tones for non-checked syllables in standard Hong Kong Cantonese, namely T1 (high level tone), T2 (high rising tone), T3 (mid level tone), T4 (low falling/extra low level tone), T5 (low rising tone), and T6 (low level tone). Their pitch contours are shown in Figure 1. In that study, a discrimination task and a production task were administered to 120 native Cantonese speakers in Hong Kong. The results confirmed the suspected merger of T2 and T5 since a significant number of subjects were noted to fail to contrast the two tones in both perception and production. The dissociation between perception and production of tonal contrasts was also observed. The more interesting finding was the identification of groups of speakers whose tone production distinguished all tone categories in the language, but who failed to distinguish just one particular tonal contrast in perception. Among all the tonal contrasts, the T4/T6 contrast exhibited the strongest dissociation effect.

One may argue that in studies employing behavioral tasks such as auditory discrimination or identification, the participants’ perception involves not only processing of auditory information, but also their judgments and decisions. Judgments, however, may not necessarily reflect our ability to distinguish two auditory stimuli. For instance, a discrepancy has been reported between poor performance in a behavioral task of musical pitch discrimination and subcortical neurophysiological response at magnitudes indicative of pitch distinction in a group of native speakers of Mandarin Chinese [33]. Referring to the near-merger phenomenon, it is possible that the listener’s failure to distinguish two tones as indicated by a decision may be accompanied by distinctive responses to the same stimuli at the brain level. Hence, a far more accurate assessment of an individual’s ability to perceive sensory differences is to observe his or her neural responses.

Among currently available neuroimaging methods, the event-related potential paradigm (ERP) is uniquely suitable for investigating the issue in hand. Of the various ERP components associated with different stages of auditory/phonological processing, the mismatch negativity (MMN), which reflects a pre-attentive comparison process in passive oddball paradigms and can be elicited independently of bias of any experimental task or participant’s strategy ([34], [35], see also [36] for a revised view of MMN in relation to the role of attention in its generation), has been used extensively to examine the sensitivity of individuals to speech sound contrasts, including place of articulation, voicing, and vowel, as well as changes in pure tones, complex tones and repetitive tone patterns (see [37] for review). Studies successfully elicited MMN to tonal contrasts among native speakers of Mandarin [38] and Cantonese [39] have been reported. Both studies found greater MMN amplitude and shorter MMN peak latency in response to tonal contrasts that are larger in height and contour. Furthermore, a shorter peak latency of P3a, a positive-going ERP component following MMN reflecting automatic attention switching, was observed to result from greater height differences [39].

In the present investigation, participants who could discriminate all contrastive tones in Cantonese in production and perception as well as those who could produce all tones distinctively but specifically failed to discriminate T4 and T6 in perception were invited to take part in an ERP study with a passive oddball paradigm. The two groups of participants were presented with lexical (i.e. meaningful) and nonlexical (i.e. meaningless) syllables with T1/T6 and T4/T6 contrasts. The T4/T6 contrast behaviorally distinguished the two participant groups, while the T1/T6 contrast could be discriminated by both. Additionally, the T1/T6 contrast was chosen as the same condition was examined previously [39]; this would allow direct comparison between this study and previous results. Nonlexical syllables were employed although we could not properly evaluate the proposal regarding the dynamic interaction between speech perception and production as a function of familiarity [7] in the case of Chinese. The reason is that it is not possible to elicit production of nonlexical syllables without involving perception of the same items, for instance in a repetition task, and reading aloud nonlexical characters would always result in production of existing syllables [40]. Nevertheless, assessing perception of nonlexical syllables using a passive oddball paradigm was still highly informative, as it would reveal whether those participants with good perception of all tonal contrasts of lexical syllables could indeed discriminate the same contrasts in unfamiliar contexts devoid of meaning.

If dissociation between good production and poor perception among normal speakers is a genuine phenomenon, then ERP results associated with lexical syllables would align with behavioral observations, that is, presence of MMN in the T1/T6 contrast exhibited by both participant groups, and divergent findings in terms of absence of MMN in the T4/T6 contrast for the group with tone perception and production dissociation but presence of MMN for participants without dissociation. Moreover, the T1/T6 contrast would elicit higher MMN amplitude, shorter MMN and P3a peak latencies than T4/T6, based on previous findings [38], [39]. For perception of nonlexical syllables, reduced MMN amplitudes and longer latencies are expected compared with lexical syllables, in light of previous findings of lower MMN amplitudes and later peak latencies induced by pseudo words or low frequency words than real or higher frequency lexical items [41], [42], [43], [44]. The most interesting observation would be to see if brain responses to the T4/T6 contrast of nonlexical syllables in individuals without dissociation would be similar to those of lexical syllables. In other words, the aims of this study were to confirm the near-merger phenomenon using neurophysiological measures, which are arguably more sensitive than behavioral measures, and to understand the theoretically intriguing and challenging dissociation pattern between good speech production and poor perception through contrasting perception of lexical and nonlexical syllables. In addition, since participants of both study groups are normal speakers, individual differences were also explored to account for the differential perceptual sensitivity to particular tonal contrasts.

## Methods

### Ethics Statement

Informed written consent was obtained from all participants before the study began. The experiment was performed in accordance with the Declaration of Helsinki with approval of the University of Hong Kong Human Research Ethics Committee for Non-Clinical Faculties.

### Participants

Forty native speakers of Cantonese with no history of neurological disorder or known hearing deficits were recruited using the screening procedures in [31], [32]. According to the Edinburgh Handedness Inventory [45], there were two left-handers and two ambidextrous participants in the control group, one with left bias and one with right bias. In the dissociation group, there were one left-hander and three ambidextrous participants, two with right bias and one with left bias. The other participants were right-handers. The participants were selected on the basis of their performance in an AX discrimination task and a reading aloud task. In the perception task, the participants had to indicate on each trial whether two aurally presented segmentally identical syllables sounded the same or not by clicking a button on the computer screen. The stimuli represented 21 pairs of tonal contrasts (six of which were identical) of eight trials each. The stimulus syllables were generated from eight syllable roots *fu*, *se*, *si*, *ji*, *ku*, *po*, *ja*, *je* coupled with each of the six tones in Cantonese. Thirty-six of the 48 resultant syllables were lexical syllables in the language, while the rest were nonlexical syllables. In the rest of this paper, phonetic transcription of Cantonese is given in *jyutping*, a romanization system developed by the Linguistics Society of Hong Kong. The number in the transcription represents the tone. The 36 lexical syllables in the perception task were targets in the reading aloud task. Characters representing these syllables were embedded in two carriers in the middle and final positions of the sentence, *ngo5 ji4 ga1 duk6 ___ zi6* ‘I am now reading the _____ characters’ and *ni1 go3 zi6 hai6 ___* ‘The character is ______’, resulting in 72 trials. The speech outputs were recorded digitally, and were assessed by two native Cantonese raters with training in phonetics who were blind to the group status of the participants. The raters individually judged whether the produced tones corresponded to the target tones. The output was then classified as correct or incorrect. When a tone error occurred, the rater had to determine the lexical tone that closely matched the speech token. An intermediate form between the target tone and another tone was marked as intermediate and was classified as an incorrect response. Cases of disagreement were discussed until an agreement for each case was reached between the two raters.

All participants in this study could produce the six Cantonese tones distinctively. Half of them were also able to perceptually discriminate all tonal contrasts, henceforth the ‘control’ group. The other participants could distinguish most tonal contrasts except T4/T6, henceforth the ‘dissociation’ group. The control group (10 males) had a mean age of 33.7 years (range: 20–58 years, *SD* = 15.06); the dissociation group (9 males) had an average age of 32.2 years (range: 19–61 years, *SD* = 14.01). The participants’ performance on the three tones of interest in the present study, i.e. T1, T4, and T6, in the discrimination and production tasks is summarized in Table 1. Note that for those participants who did not produce T4 correctly 100% of the time (one participant), the errors did not involve production of T6, and vice versa for those who did not achieve 100% correct on production of T6 (eight participants).

**Table 1:** Participants’ performance in mean (*SD*) in the tone discrimination and production tasks.

	Discrimination		Production		
	T1/T6	T4/T6	T1	T4	T6
Control group	100%	100%	100%	100%	100%
Dissociation group	100%	53.75%(13.51)	100%	99.59%(1.86)	95.77%(5.92)

### Stimuli

The stimuli in this study included the lexical syllable *fu* and the nonlexical syllable *lu* carrying T1, T4, or T6, recorded by a native Cantonese female speaker. *fu* was chosen because the combinations of the syllable with each of the six distinct tones in Cantonese all resulted in existing syllables, i.e. *fu1* ‘exhale’ ‘husband’, *fu6* ‘to pay’, ‘negative’, ‘father’, *fu4* ‘to assist’; in contrast, *lu* would not form meaningful syllables with any of the three target tones. The experiment employed a 2×2×2 factorial design, i.e. syllable type (lexical vs. nonlexical syllables)×tonal contrast (T1/T6 vs. T4/T6)×T6 as deviant vs. standard stimuli, resulting in eight blocks. Each block contained 535 trials, of which 80 trials (or 15%) were deviant trials. The trials were randomized with the constraint that there would be a minimum of five and a maximum of 11 standards between consecutive deviants. Each trial lasted 1200 ms, including a syllable of 400 ms in length and an inter-stimulus interval (ISI) of 800 ms (similar in length to [46], [47]). The intensity of all syllables was normalised to 70 dB SPL using Audacity (http:/audacity.sourceforge.net/) and all syllables were aligned to have the same onset (100 ms) and vowel (300 ms) duration. This was critical for defining the divergence point–where two stimuli begin to deviate–and for identifying the MMN as MMN is highly sensitive to minor acoustic differences. The divergence point was different for the two pairs of tonal contrasts due to the difference in pitch. T1 and T6 diverged at the vowel onset (100 ms post-stimulus onset) as they have different pitch heights. In contrast, T4 resembled T6 in the early part of the pitch contour; the divergence point of T4/T6 was later than that of T1/T6 and was also slightly different for the syllables *fu* and *lu*. *fu4* and *fu6* began to diverge at 200 ms post-stimulus onset, whereas *lu4* and *lu6* at 180 ms.

### Procedure

The passive oddball task took place in the Laboratory for Communication Science in the Division of Speech and Hearing Sciences at the University of Hong Kong. The participants were seated comfortably in front of a computer screen approximately 1 m away. During the task, participants were asked to watch a silent movie played via the computer screen. Auditory stimuli were binaurally presented through headphones simultaneously. The participants were told to pay attention to the movie and ignore the sounds they hear. The task consisted of eight blocks and a two-minute rest was given between blocks. Four sequences of the eight blocks were rotated across participants. T1/T6 contrasts were presented before T4/T6 contrasts for half of the participants. For each tonal contrast, lexical syllables were presented before nonlexical syllables. Consecutive blocks of lexical or nonlexical syllables of the same tonal contrast differed in terms of which tones served as the deviant and standard, respectively. The entire experiment lasted about 100 minutes.

### Data Acquisition and Analysis

Electrocephalography (EEG) was recorded using SynAmps2 Neuroscan Inc. system (Compumedics Ltd., USA) in an electrically and acoustically shielded booth. The EEG activity was recorded from 64 silver-silver-chloride (Ag/AgCl) electrode sites (FPz, Fz, FCz, Cz, CPz, Pz, POz, Oz, FP1/2, F7/5/3/1/2/4/6/8, FT7/8, FC5/3/1/2/4/6, T7/8, C5/3/1/2/4/6, M1/2, TP7/8, CB1/2, CP5/3/1/2/4/6, P7/5/3/1/2/4/6/8, PO7/5/3/4/6/8, O1/2) arranged in an extended montage based on the International 10–20 system (using a Neuroscan 64-channel Quik-cap, Compumedics Ltd., USA). The vertex functioned as the reference and AFz served as the ground electrode. The impedance was kept under 10 kΩ whenever possible. Additional electrodes were placed above and below the left orbit and on the outer canthus of each eye to monitor electro-oculographic (EOG) activity with a bipolar recording. Continuous data were digitized at a sampling rate of 500 Hz with a bandpass of 0.05 Hz to 200 Hz. The collected raw EEG data was preprocessed with Neuroscan 4.5 software (Compumedics Ltd., USA) and FieldTrip [48]. The data were first filtered with a bandpass 1 Hz to 20 Hz for noise reduction and were then divided into trials of 1800 ms in length including an 800 ms interval before the stimulus onset. Extreme trials–trials with an amplitude larger than ±300 µV–were then removed before entering all trials into Independent Component Analysis (ICA). The purpose of the ICA was to identify any components resembling eye blinks, horizontal eye movements, noisy channels and other focal artefacts. The identified components were then mathematically removed from the data and signals were reconstructed based on the remaining components. After ICA, each channel was baseline corrected using the pre-stimulus 800 ms interval and was re-referenced to the mean mastoids to remove any lateral bias [46], [47]. Trials with artefacts that exceeded 100 µV, trends greater than 75 µV, abnormal distributions or improbable data exceeding 5 *SD*s were also rejected. This procedure removed a total of 147 trials (or 0.77% of all trials) in the control group, and 516 trials (2.67%) in the dissociation group. The remaining trials were sorted into (i) deviant (per tone and syllable-type), (ii) standard-before-a-deviant (per tone and syllable-type), and (iii) standard preceding each standard-before-a-deviant (per tone and syllable-type), following [46], [47]. Subtraction of (ii) from (i) rendered true difference waves, while subtraction of (iii) from (ii) resulted in dummy waves. Comparisons with the dummy difference waves were to reduce the chances of identifying random fluctuations as MMN.

In this paper, we focused on data from the four blocks, *fu1*/*fu6*, *fu4*/*fu6*, *lu1*/*lu6*, *lu4*/*lu6*, with T6 as the deviant. Statistical differences between the true and dummy MMN waves were assessed by a non-parametric cluster-based permutation test [49]. The test first identifies sampling points with t-statistic exceeding a critical threshold (*p*<.05, two-tailed). Clusters were then formed by connecting significant sampling points on the basis of spatial and temporal adjacency. This was done separately for sampling points with positive and negative t-values. The maximum cluster-level test statistics (the sum of all individual t-values within a cluster) were then computed to generate permutation distributions, one for positive cluster and one for negative cluster, based on 10,000 random partitions. The significance of a cluster was determined by whether it fell in the highest or the lowest 2.5^th^ percentile of the corresponding distribution. The cluster-based permutation tests were carried out on each block for each participant group to identify significant MMN and P3a components.

A more standard approach of analysis was also taken. Mixed model ANOVAs were conducted to compare the peak latency and average amplitude of MMN and P3a (i.e. amplitude difference between true difference waves and dummy waves), respectively, at the FCz electrode where the strongest effects across experimental conditions were found. For MMN, the latency was based on the most negative peak during the time window of 100–250 ms post-divergence point. For P3a, the latency was identified according to the most positive peak following the individual MMN peak. For both components, the average amplitude was computed of a 100 ms time window centered on the MMN and the P3a peaks, respectively, of the relevant grand averaged waves (similar to [50]). Greenhouse-Geisser correction was applied when appropriate to protect against Type I errors.

## Results

### Cluster-based Permutation Test

**Figure 2:**
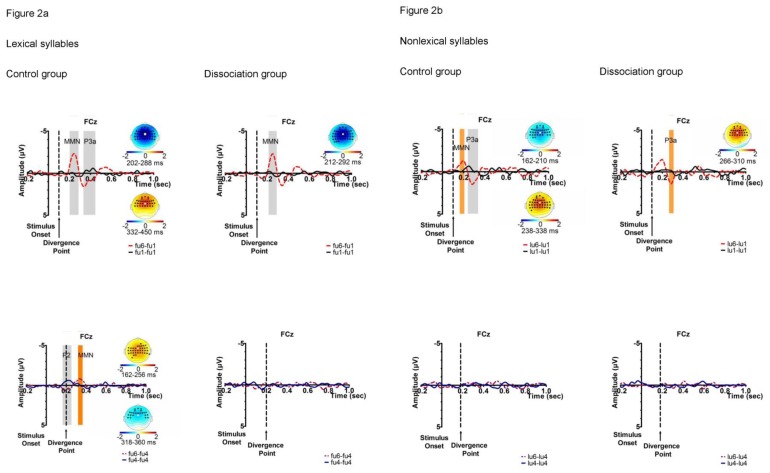
Panel A. Tonal contrasts of lexical syllables. Panel B. Tonal contrasts of nonlexical syllables. *Note*. Grand-averaged difference waves and dummy waves at the FCz electrode for illustration, with significant clusters (statistically significant ones in grey and clusters uncorrected for multiple comparisons in orange) considered as MMN or P3a components and their corresponding topographs (significant electrodes in black and FCz in white).

The results of the cluster-level permutation test shown in Figure 2 revealed several significant clusters in different conditions in the two participant groups. Note that significant clusters represent sampling points with spatial and temporal adjacency; they are superimposed on grand averaged waves of FCz mainly for the purpose of illustration. For lexical syllables *fu1*, *fu4*, and *fu6*, the contrast between *fu1* and *fu6* elicited a significant negative cluster with a time window during 102–188 ms (post-divergence point unless specified otherwise) typical of an MMN (sum-T = −7.92, *p*<.01), and a marginally significant positive cluster (232–350 ms; sum-T = 5.04, *p* = .060) in the control group, which can be considered a P3a. The dissociation group demonstrated one significant negative cluster in a typical time window of MMN (112–192 ms; sum-T = −7.12, *p*<.01). In the *fu4*/*fu6* comparison, the control participants also showed an early positive cluster (from 38 ms pre-divergence point to 56 ms post-divergence point; sum-T = 6.74, *p* = .010). For nonlexical syllables *lu1*, *lu4*, and *lu6*, the only significant cluster is a positive one exhibited by the control participants in the *lu1*/*lu6* contrast (138–238 ms; sum-T = 6.92, *p* = .012), which is identified as a P3a. The scalp distributions of all these clusters are consistent with our interpretation (see Figure 2).

Additionally, we observed four significant clusters, three negative and one positive, that are worth mentioning, although they did not survive control of the critical false alarm (FA) rate of.05– negative clusters (MMN) in the *fu4*/*fu6* contrast (118–160 ms) and *lu1*/*lu6* (62–110 ms) by the control group, and one positive cluster (P3a) exhibited by the dissociation group also in *lu1*/*lu6* (166–210 ms). As the first negative cluster (32–114 ms) in the *lu1*/*lu6* comparison of the dissociation group did not exhibit the typical MMN typography in terms of insignificant effects in the frontal electrodes, it would not be interpreted as such. No significant cluster was found in the comparisons between *fu4* and *fu6* by the dissociation group, and *lu4*/*lu6* by either participant group.

To summarize, statistically reliable MMN and P3a clusters were observed only in the comparisons of T1/T6. The control participants exhibited both an MMN and a P3a in the condition with lexical syllables and a P3a in response to nonlexical syllables. In contrast, the dissociation group only showed an MMN in the comparison involving lexical syllables.

### ANOVA Test

For the mixed model ANOVA analyses, a three-way (participant×lexicality of syllable×tonal contrast) ANOVA for the MMN latency and a two-way (participant×lexicality of syllable) ANOVA for the P3a latency of T1/T6 of individual peaks were carried out. The T4/T6 contrast was not included in the P3a analysis since no significant relevant cluster (corrected or not) emerged in the permutation test. For comparisons of averaged amplitudes across experimental conditions, a 100 ms time window was centered on the averaged MMN peaks from the control *fu6*/*fu1*, dissociation *fu6*/*fu1*, and control *lu6*/*lu1* conditions at 132 ms, one on the MMN peak of *fu4*/*fu6* at 136 ms, and one on the averaged P3a peaks from the control *fu6*/*fu1*, control *lu6*/*lu1*, and dissociation *lu6*/*lu1* conditions at 202 ms. Similar to the peak latency analysis, three-way and two-way ANOVAs were conducted for MMN and P3a, respectively.

**Figure 3:**
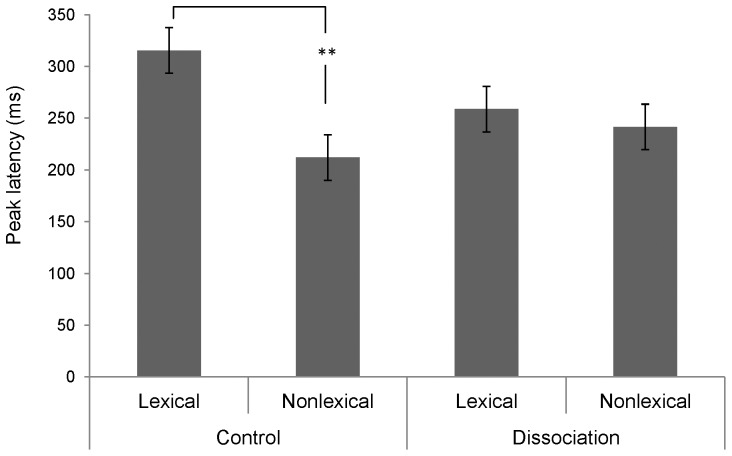
P3a peak latency (post-divergence point) of different participant group and syllable type conditions. *Note*. Lexical = lexical syllable, nonlexical = nonlexical syllable, control = control group, dissociation = dissociation group, ** = *p*<.01.

The peak latencies of MMN in different conditions are shown in Table 2. The three-way ANOVA did not find any significant main effects or interactions (*p*>.18). The analysis of P3a peak latency (post-divergence point) found a significantly main effect of lexicality of syllable, *F*(1, 38) = 6.68, *p*<.05, η^2^ = .15, and a marginally significant interaction between participant group and lexicality of syllable, *F*(1, 38) = 3.41, *p* = .072, η^2^ = .08. The peak latency of nonlexical syllables (*M* = 226.75 ms, *SE* = 15.68) was earlier than lexical syllables (*M* = 287.05 ms, *SE* = 15.22). The control group exhibited an earlier P3a to nonlexical than lexical syllables, *t*(19) = 3.11, *p*<.01; no comparable difference was seen in the dissociation group (*p*>.60)(see Figure 3).

**Table 2:** MMN peak latencies (post-divergence point) by participant group in different syllable and tonal contrast conditions.

Participant group	Syllable	Tonal contrast	Mean latency in ms (SD)
Dissociation	Lexical - *fu*	T1/T6	161.80 (46.44)
		T4/T6	166.70 (50.75)
	Nonlexical - *lu*	T1/T6	161.90 (56.96)
		T4/T6	164.40 (35.93)
Control	Lexical - *fu*	T1/T6	161.30 (51.84)
		T4/T6	158.30 (57.32)
	Nonlexical - *lu*	T1/T6	149.10 (60.13)
		T4/T6	137.50 (35.50)

**Figure 4:**
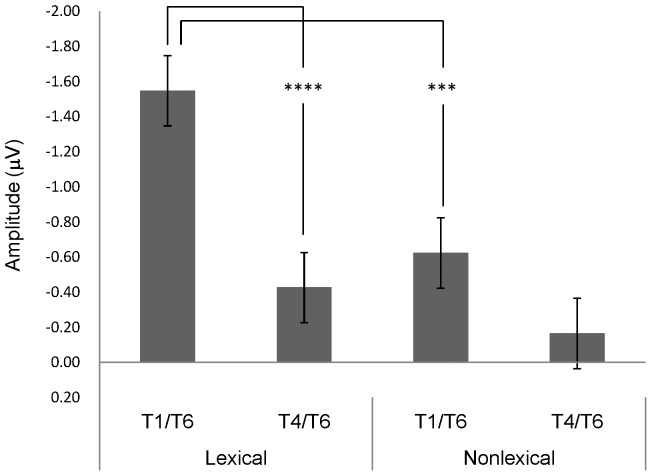
Average MMN amplitude of different syllable type and tonal contrast conditions. *Note*. T1/T6 = contrast between T1 and T6, T4/T6 = contrast between T4 and T6, lexical = lexical syllable, nonlexical = nonlexical syllable, *** = *p*<.005, **** = *p*<.001.

For average amplitude of MMN, only significant main effects of lexicality of syllables, *F*(1, 38) = 9.98, *p*<.005, η^2^ = .21, and tonal contrast, *F*(1, 38) = 16.26, *p*<.001, η^2^ = .30, were found. Lexical syllables (*M* = −.99 µV, *SE* = .15) elicited a stronger MMN response than nonlexical syllables (*M* = −.39 µV, *SE* = .13); T1/T6 (*M* = −1.09 µV, *SE* = .15) resulted in higher MMN amplitude than T4/T6 (*M* = −.30 µV, *SE* = .14). The interaction between syllable and tonal contrast was marginally significant, *F*(1, 38) = 3.43, *p* = .072, η^2^ = .08 (see Figure 4). Pairwise comparisons showed that the difference between T1/T6 and T4/T6 of lexical syllables was significant, *t*(39) = −3.99, *p*<.001; however, that of nonlexical syllables was not (*p*>.05). In addition, the contrast between lexical and nonlexical syllables of T1/T6 was also reliable, *t*(39) = −3.27, *p*<.005, but that of T4/T6 was not (*p*>.27). No significant effects, participant or lexicality of syllable, were observed in average P3a amplitude (*p*>.7).

In summary, the ANOVA analyses of amplitude and peak latency at FCz did not find main differences between the two participant groups. An earlier P3a latency to nonlexical syllables than lexical syllables was observed; moreover, the two groups differed in terms of a quicker response to nonlexical than lexical syllables only in the control group. Lexical syllables and T1/T6, respectively, elicited stronger MMN responses than nonlexical syllables and T4/T6. While the MMN response to T1/T6 was stronger than that to T4/T6 of lexical syllables, no such difference was found for nonlexical syllables.

## Discussion

This study examined the perplexing dissociation between good production and poor perception of Cantonese tones, i.e. the tone near-merger phenomenon, using ERP measures from a passive oddball paradigm and presenting both lexical and nonlexical syllables. We also attempted to understand possible perceptual and/or cognitive differences between participants with and without dissociation as reflected in the MMN and P3a amplitudes and peak latencies.

The results of the cluster-based permutation test revealed statistically reliable clusters corresponding to the MMN and P3a to the T1/T6 contrast and a MMN cluster (uncorrected) to T4/T6 of lexical syllables in the control participants. The presence of MMN and P3a to the T1/T6 contrast is consistent with previous findings [39]. In contrast, the dissociation group exhibited only a reliable MMN cluster to T1/T6, and no other cluster (corrected or uncorrected) in the same conditions. The differential neural responses of the latter group to the two tonal contrasts were consistent with the dissociation pattern of behavioral measures, and thereby confirmed the tone near-merger phenomenon.

In the conditions with nonlexical syllables, the T1/T6 contrast elicited a reliable P3a and an (uncorrected) MMN cluster in the control group, as well as an uncorrected P3a in the dissociation group. This pattern demonstrated that brain responses to nonlexical syllables were generally weaker than lexical syllables for all participants. These cluster-level permutation results were compatible with the significant main effect of lexicality of syllables in the ANOVA test, where higher MMN amplitudes were observed in lexical than nonlexical syllables. The main effect of tonal contrast, T1/T6 vs. T4/T6, was largely due to indistinctive T4/T6 contrasts of lexical syllables in the dissociation group and of nonlexical syllables in both participant groups. The marginally significant interaction between syllable status and tonal contrast was related to the significant difference between lexical and nonlexical syllables for the T1/T6 contrast and its absence for the T4/T6 contrast (see Figure 4). Focusing on the control participants, the relative strength of the MMNs across experimental conditions, i.e. statistically reliable MMN cluster in *fu1*/*fu6*, significant (but uncorrected) clusters in *lu1*/*lu6* and *fu4*/*fu6*, and null response in *lu4*/*lu6*, suggests that the good performance on tonal discrimination in the behavioral task and the MMN response to the T4/T6 contrast of lexical syllables are not simply driven by distinctive tone perception.

Contrary to expectations, no effects of lexical vs. nonlexical syllables or tonal contrasts were obtained in the analysis of MMN peak latency. This is evident in the dissociation group as illustrated in Table 2. Moreover, the control group showed a tendency of shorter MMN peak latency to nonlexical than lexical syllables, both in the case of T1/T6 (12.2 ms) and T4/T6 (20.8 ms). Further consideration of this point is given when we discuss the results of P3a latency.

Regarding the P3a component, its occurrence (either as a reliable or uncorrected cluster) does not seem to be dependent on its concomitance with an MMN. As seen in Figure 2, an MMN may not be followed by a P3a as in lexical syllables, T1/T6 of the dissociation group and T4/T6 of the control group. Likewise, P3a may not be preceded by an MMN as in nonlexical syllables T1/T6 of the dissociation group. This double dissociation between the two ERP components suggests that they may reflect processes of different cognitive levels, where MMN is triggered by change or deviant detection and is not particularly sensitive to manipulation of attention allocation, while P3a may reflect a “higher level event-detection process” (p. 146, [50]) and is subject to top-down control of attention switching, such as predictability of occurrence of deviant stimuli [51] (see also [52], [53]). While the ANOVA results of P3a amplitudes at FCz did not demonstrate effects of lexicality of syllable or participant group, the cluster-based permutation test revealed qualitative differences between the two groups, in terms of presence of P3a in the control group versus its absence in the dissociation group to the T1/T6 contrast of lexical syllables, and a statistically reliable P3a among the control participants versus a significant but uncorrected P3a among the dissociation participants to the same tonal contrast of nonlexical syllables.

The P3a peak latency, on the other hand, showed a significant main effect of syllable status and a marginally significant interaction between participant group and lexicality. The control participants exhibited a shorter P3a latency to nonlexical syllables of the T1/T6 contrast than lexical syllables (see Figure 3). The contrast in P3a latency in relation to the lexicality of syllable is similar to the trend of MMN latency we mentioned earlier. Only control participants showed shorter MMN latency to nonlexical than lexical syllables, regardless of tonal contrast. These observations are at odds with previous findings that participants responded faster and more strongly to familiar than unfamiliar stimuli, or lexical than nonlexical syllables. We examined the pitch difference in each tonal contrast by lexicality condition to see if that might provide a possible explanation for the observation. We found a difference of 100.4 Hz between nonlexical syllables *lu1* and *lu6* (284.7 Hz vs. 184.3 Hz), 84 Hz between lexical syllables *fu1* and *fu6* (254.9 Hz vs. 170.9 Hz), 4.1 Hz between *lu6* vs. *lu4* (184.3 Hz vs. 180.2 Hz), and 7.7 Hz between *fu6* vs. *fu4* (170.9 Hz vs. 178.6 Hz). Although it seemed plausible that a larger pitch contrast between the lexical and nonlexical stimuli of the T1/T6 contrast could have induced a faster neural response in the form of shorter MMN (albeit only a tendency) and P3a latencies [54], the proposal has difficulty explaining the trend of greater MMN latency difference exhibited by the control group in the T4/T6 contrast when the pitch difference between the lexical and nonlexical stimuli is small and in the opposite direction. Moreover, the lexicality effect was only demonstrated in the control group. These render the account of pitch difference *alone* for earlier MMN and P3a peak latencies rather untenable.

One of the objectives of the present study, in addition to confirming the near-merger phenomenon, is to understand its underlying mechanism; in other words, why some normal speakers would no longer be able to distinguish certain tonal contrast despite their ability to produce them, while other normal speakers remain capable of distinguishing all tones in both perception and production. Here we put forth a speculative account. We have noted earlier that the controls differed from the participants with dissociation regarding P3a. While the control participants consistently exhibited a reliable P3a cluster in the T1/T6 contrast of lexical and nonlexical syllables, these conditions failed to elicit a P3a reliably in the dissociation group. Furthermore, the effect of lexicality on P3a peak latency was only evident in the control participants. The P3a has usually been described as an index of involuntary attention switching following change/deviant detection indicated by an MMN. However, the dissociation in occurrence between the two components (e.g. [50]) has led to the proposal that they are associated with processes at different cognitive levels, as mentioned before. The MMN is stimulus-driven and does not seem to be affected by manipulation of predictability of occurrence of the deviant stimulus, whereas the P3a may reflect a higher-level event detection process [50] and its amplitude can be reduced if the occurrence of the deviant is predictable, suggesting the influence of top-down control [51]. More interestingly, the P300, of which P3a is a subcomponent responsive to presentation of task irrelevant stimulus, has been said to be negatively correlated with an individual’s cognitive capability and positively related to one’s speed of attention allocation ([55]; see also [54] for a review). Shorter P300 latencies are associated with higher cognitive performance, such as recognition memory performance in a list learning task [56], auditory short-term memory [54], and total memory score in a digit span memory task [57] (but see [58] for an observation in the opposite direction in a memory task with varying task difficulty). We recognize that it is far from clear as to which cognitive process(es) P300 (or P3a) is correlated with, we tentatively propose that P3a amplitude and/or latency may reflect one’s level of functioning in top-down control of attentional shifting, auditory attention or memory, and that higher capability in these cognitive domains may partly determine an individual’s auditory perceptual ability.

A crucial question in the current findings that must be addressed is the reduced and diminished sensitivities of the control group and the dissociation group, respectively, to the T4/T6 contrast, which represents a small, if not the smallest, auditory difference in the Cantonese tonal system, while distinctive production of these tones is largely preserved. We propose that the degraded sensitivity or indistinctive perception of T4/T6 among normal Cantonese speakers is a consequence of top-down processing in language communication, in which rich contextual information from all linguistic levels including semantic, syntactic and pragmatic allows one to continuously make predictions about upcoming linguistic information. Therefore, recognition of lexical items in spoken language does not necessarily depend on a complete analysis of acoustic signals. In other words, word recognition is not solely a bottom-up process.

Many models of speech perception recognize the integration between top-down and bottom-up processing (e.g. [15], [59], [60], [61]); they vary in the stage at which the two types of processes interact. Some of the earliest demonstrations of top-down processing include the phoneme restoration effect [62], the well-known Ganong effect [63], and the contextual effect on isolation point of words in a Gating paradigm [64] (see [65] for a review). Top-down perception of phonemes can also be influenced by input from another modality, i.e. visual speech reflecting movements of facial articulators [66]. As visual speech precedes auditory input, speakers are able to make online prediction of auditory signals based on articulatory gestures. Indeed, simultaneous presentation of auditory and visual (AV) speech was found to facilitate speech perception resulting in shorter latencies of N1 and P2 components, and that the more salient and predictable the visual speech was, i.e. [pa] vs. [ka], the greater the facilitation.

The findings of application of prior knowledge to bottom-up speech processing on the part of the listener [66] are reinforced by the results of the relative timing between top-down and bottom-up processes in speech perception [67]. In that study, the extent of prior knowledge and the quality of acoustic signal were manipulated. The former was provided by written material which might be a string of “x” or a word, which might be matched or mismatched with the following spoken word of varying degrees of degradation of speech signal. Using concurrent EEG and magnetoencephalography (MEG) recordings, Sohoglu et al. measured the neural responses in the left inferior frontal gyrus (LIFG) and the left superior temporal gyrus (LSTG) assumed to underlie top-down and bottom-up processing, respectively, over the time windows of 90–130 ms, 180–240 ms, 270–420 ms, 450–700 ms post-stimulus onset. They found that effects of prior knowledge were evident from the earliest time window in the LIFG, indicating that top-down predictions were generated once initial phonetic detail (even degraded) was available.

Our results of higher MMN amplitudes to lexical than nonlexical syllables can be considered compatible with top-down processing; however, to account for the observation of lower or even a lack of sensitivity to small tonal contrasts among normal native speakers requires us to further propose that constant interaction between top-down and bottom-up processing may mean that acoustic input often does not undergo complete analysis for speech recognition, and a consequence of that is an individual’s sensitivity to speech sound distinctions, particularly of small differences (i.e. T4/T6 contrast), may be weakened overtime. The extent of the reduction would partly be dependent on the individual’s cognitive capability in the domains of auditory memory, auditory attention, or attentional switching in general. If P3a can be taken as an index of such abilities as previous literature has suggested, this may imply that the participants in the control group are stronger in these cognitive areas than those in the dissociation group, and this difference may affect their ability to maintain distinctive perception of T4 and T6 in the language. In proposing this account, we assume that under certain circumstances and for certain individuals, speech recognition can be solely driven by top-down predictions.

Finally, two aspects of the results are briefly considered for future study. The cluster-based permutation test revealed an early positive going component over the central frontal region larger over the right hemisphere exhibited by the control group in the T4/T6 contrast of lexical syllables. We refer to it as P2. This component was mentioned in a number of studies investigating auditory discrimination of stimuli varying in pitch, duration, or tone patterns [68], [69], [70]. The exact cognitive function(s) that this P2 is related to is unclear. It has been suggested that it reflects stimulus classification [70], an attention-modulated process in auditory discrimination [69], as well as an index of activation of long-term sensory memory correlated with the size of short-term memory set [68]. However, in stark contrast with our observation, the P2 reported in those studies was elicited by the standard or more frequent stimulus. Moreover, the significant P2 cluster in our results began before the divergence point. We, therefore, have no explanation for its occurrence and would be interested in seeing whether the observation would be replicated.

The alert reader may also recall that in the tone discrimination task we used for identifying appropriate participants, the materials included both lexical and nonlexical syllables. Contrasts of lexical vs. lexical, lexical vs. nonlexical, and nonlexical vs. nonlexical syllables were presented. The perfect performance achieved by the control participants must therefore have included trials consisted of nonlexical syllables only. Given this, one may raise a question about the discrepancy between good distinction of tonal contrasts of nonlexical syllables and an absence of a difference in neural response to the *lu4*/*lu6* contrast. A search through the stimulus set showed that there were no trials involving a nonlexical syllable pair of the T4/T6 contrast. Further extension of the current work should examine all tonal contrasts realized in all combinations of lexical and nonlexical syllables.

In conclusion, our findings of significant MMN and P3a to the T1/T6 contrast exhibited by the control participants are consistent with [39]. The absence of MMN to T4/T6 in the Dissociation group has confirmed the tone near-merger phenomenon previously based on behavioral performance. The contrast in strength of MMN response to lexical and nonlexical syllables indicates top-down processing in speech perception. To account for the difference between the two participant groups in terms of dissociation between poor perception and good production, we tentatively attribute it to their different cognitive capabilities in attentional shifting, auditory attention and memory indexed by P3a amplitude and/or latency, which may influence their ability to maintain sensitivity to small tonal contrasts, and to incomplete bottom-up analysis of acoustic input due to constant top-down predictions in normal speech processing.
